# Controversies and Recent Advances in Hematopoietic Cell Transplantation for Follicular Non-Hodgkin Lymphoma

**DOI:** 10.1155/2012/897215

**Published:** 2012-10-11

**Authors:** Abraham S. Kanate, Mohamed A. Kharfan-Dabaja, Mehdi Hamadani

**Affiliations:** ^1^Myeloma and Lymphoma Service, Osborn Hematopoietic Malignancy and Transplantation Program, West Virginia University, Morgantown, WV 26506, USA; ^2^Blood and Marrow Transplantation, Moffitt Cancer Center, Tampa, FL 33612, USA; ^3^Division of Hematology and Oncology, West Virginia University, P.O. Box 9162, 1 Medical Center Drive, Morgantown, WV 26506, USA

## Abstract

Commonly designated as an indolent non-Hodgkin lymphoma, follicular lymphoma (FL) presents with striking pathobiological and clinical heterogeneity. Initial management strategies for FL have evolved to involve combination chemoimmunotherapy and/or radio-immunoconjugates. Unfortunately even with the best available nontransplant treatment, which nowadays results in higher frequency of response, FL remains incurable. Although considered a feasible therapeutic option, the use of hematopoietic cell transplantation (HCT) remains controversial. The appropriate timing, graft source, and intensity of HCT conditioning regimens in FL are often matters of debate. Herein we review the available published data pertaining to the use of autologous or allogeneic HCT in patients with FL across different stages of the disease, discuss major recent advances in the field, and highlight avenues for future research. The current literature does not support a role of HCT for FL in first remission, but in the relapsed setting autologous HCT remains appropriate for patients with early chemosensitive relapses, while allogeneic transplantation remains the sole curative modality for this disease, in relatively younger patients without significant comorbidities.

## 1. Introduction

Follicular lymphoma (FL) is the second most common type of non-Hodgkin lymphoma (NHL) in the western hemisphere accounting for 22% of all cases [1]. The median age at diagnosis is generally in the 6th decade, with a slight female preponderance. Being an indolent lymphoma, the disease course of FL is one of remissions and relapses with conventional chemoimmunotherapies followed not infrequently by development of resistance and/or transformation into a more aggressive histology. A subset of FL patients has a more aggressive clinical course, with approximately 15% mortality at 2 years resulting from progressive or transformed disease [2]. While clinical prognostic systems such as FL international prognostic index (FLIPI) are good in estimating overall survival (OS) [3, 4], they have limited predictive value in identifying patient groups that may (or may not) benefit from aggressive initial therapy. Management strategies include surveillance, combination chemoimmunotherapy, radio-immunotherapy, and autologous or allogeneic hematopoietic cell transplantation (HCT). The addition of rituximab to conventional chemotherapy regimens has resulted in improved progression-free survival (PFS) and OS [5–7] in several studies. 

Despite improved outcomes achieved with incorporation of monoclonal antibodies, namely, rituximab, or introduction of radio-immunoconjugates, namely, iodine I-131 tositumomab or ibritumomab tiuxetan, FL remains incurable. The role and timing of HCT in the management of FL is a controversial issue. While high-dose therapy (HDT) and autologous HCT (auto-HCT) has low treatment-related mortality (TRM) and morbidity, disease relapse remains a major concern. Myeloablative (MA) allogeneic HCT (allo-HCT) is a potentially curative modality; however, it is often associated with prohibitive TRM, particularly in more frail patients. Factors to be considered while assessing patients' eligibility for HCT include but are not limited to patient- and disease-related characteristics, optimal timing of HCT, type of HCT (autologous versus allogeneic), and selecting intensity of preparative regimens (MA or reduced-intensity conditioning (RIC)) in case an allograft is pursued [8, 9]. 

Herein we review the available published data pertaining to the role and optimal timing of HCT in patients with FL. To identify relevant publications, PubMed and Medline (the Web sites developed by the National Center of Biotechnology Information at the National Library of Medicine of the NIH), were searched using the search terms “follicular lymphoma” and “transplantation” limited to “English language,” and a publication date of 1992 or later. In addition to the online database search, a manual search of the reference lists of reviews and included articles was conducted. Papers that did not include FL patients or the ones that included fewer than 25 FL patients were excluded. Also excluded were editorials, letters to the editor, reviews, consensus conference papers, practice guidelines, and laboratory studies with no clinical correlates. National or international meetings' abstracts (American Society of Hematology, American Society of Blood and Marrow Transplantation, American Society of Clinical Oncology, European Hematology Association, and European Group for Blood and Marrow Transplantation) from January 2010 onwards and http://www.clinicaltrials.gov/ were searched to identify important ongoing trials. The goal of the paper is to critically analyze the current data pertaining to HCT in FL, in order to provide practical recommendation about the preferred graft source, conditioning regimen intensity, optimal timing, and the role of this modality in FL.

## 2. Role of Transplantation for FL in First Remission

Several studies have explored the use of HCT as consolidation after initial chemotherapy for FL, with the ultimate goal of improving the depth of response, disease control, and possibly OS.

### 2.1. Autologous HCT for FL in First Remission

Single center data from Dana-Farber Cancer Institute (DFCI), demonstrating prolonged disease-free survival in approximately 40% of FL patients undergoing purged bone marrow autografts, provided preliminary evidence for auto-HCT as consolidation for FL in first remission [10]. 

Four-randomized-controlled trials (RCT) have evaluated the role auto-HCT as consolidation for FL in first remission (Table 1) [11–14]. One German (German Low Grade Lymphoma Study Group (GLSG)) and two French (Groupe d'Etude des Lymphomes de l'Adulte (GELA) Groupe Ouest-Est des Leucémies et Autres Maladies du Sang (GOELAMS)) cooperative group studies randomized newly diagnosed, younger (≤60 years), advanced stage FL patients to receive consolidation with auto-HCT or interferon maintenance, after first-line chemotherapy with CHOP (cyclophosphamide, doxorubicin, vincristine, and prednisone) or CHOP-like regimens [11–13]. As shown in Table 1, a significant PFS benefit was demonstrated in favor of auto-HCT in the GLSG and GOELAMS trials, but not in the GELA protocol. To date no OS benefit has been reported in any published study. Despite a relatively low TRM after autografting in the GLSG trial, this modality, however, was associated with a significantly higher incidence of secondary hematological malignancies (3.8% versus 0%, *P* = 0.02) [11, 15]. Similarly, significantly higher frequency of second malignancies was also seen in the GOELAMS study. A major limitation of these three trials is that they were conducted in the prerituximab era, hence questioning the applicability and relevance of these results in current practice. Interestingly, the PFS of FL patients receiving rituximab-based 1st line chemoimmunotherapy in contemporary cooperative group trials is roughly similar to the PFS reported in auto-HCT arm of GELA and GLSG studies [6, 7, 16].

To address the role of auto-HCT in upfront consolidation of FL in the rituximab era, the Gruppo Italiano Trapianto di Midollo Osseo/Intergruppo Italiano Linfomi (GITMO/IIL) trial compared chemoimmunotherapy with R-CHOP to rituximab supplemented HDT and auto-HCT. While rates of complete remission (CR), molecular remission, and event-free survival (EFS) were significantly better with Auto-HCT, no difference in OS was seen. A trend towards more secondary myelodysplasia/acute myeloid leukemia (sMDS/AML) was observed in the HDT arm, albeit not statistically significant (6.6% versus 1.7%; *P* = 0.111). Lack of survival benefit, despite better disease control in the auto-HCT arm, is likely due to subsequent salvage of patients relapsing after R-CHOP alone with an autograft in second (or later) remission, among other reasons [17]. Two recently published meta-analyses of aforementioned clinical trials confirmed the PFS benefit with autografting of FL patients in first remission, but no benefit in OS was described [18, 19]. 

In view of recent advances in the treatment of patients with newly diagnosed FL, including strategies such as consolidation with radio-immunotherapy [20], rituximab maintenance [21], and/or rituximab retreatment [22], routine use of autologous transplantation as consolidation in first remission for patients with FL cannot be recommended, especially as the latter is associated with development of secondary malignancies without a benefit in OS. 

### 2.2. Allogeneic HCT for FL in First Remission

Allo-HCT offers several advantages such as a lymphoma-free graft, and the immunologic graft-versus-lymphoma (GVL) effect mediated by alloreactive donor T cells. It is a potentially curative treatment modality for patients with FL, who would be otherwise incurable with conventional chemo-immunotherapy or auto-HCT. However, there are no randomized controlled data available to support allografting in chemosensitive FL patients in first remission. Limited single-institution data are available for allo-HCT in a small subset of high risk FL patients with primary refractory disease, despite multiple treatment attempts [23, 24]. Such high-risk FL patients with primary refractory disease can be considered for an allo-HCT, ideally within the context of a clinical trial. At our institution, refractory FL patients are offered allo-HCT as part of an ongoing prospective study evaluating the role of pharmacokinetically guided reduced-toxicity conditioning allo-HCT for refractory aggressive lymphomas (http://www.clinicaltrials.gov/, NCT01203020).

## 3. HCT for Relapsed FL 

Although the majority of FL patients respond to initial therapy, the vast majority of such patients eventually experience disease progression. HCT, autologous or allogeneic, is often considered in patients with relapsed disease, particularly after multiple lines of therapies. The role, optimal timing, and preferred transplant modality (autologous versus allogeneic) in the relapsed setting remain a matter of controversy.

### 3.1. Autologous Transplantation for Relapsed FL

Auto-HCT has long been available for patients with relapsed chemosensitive disease. Early single-institution, retrospective studies showed encouraging data for patients with relapsed disease [25, 26]. Large prospective trials comparing auto-HCT with chemotherapy are lacking, adding to the existing controversy about the role of this treatment modality in relapsed FL. The European Blood and Marrow Transplant (EBMT) group reported the only RCT in this setting (CUP trial). The CUP trial compared chemotherapy alone to chemotherapy followed by either unpurged or purged autografts. This trial was closed early because of poor accrual. Notwithstanding the small number of patients that was randomized (*n* = 89), the trial showed a significant PFS and OS benefit following HDT [27]. There was no reported difference in outcomes of purged compared to unpurged autografts. However, since this trial was conducted in the pre-rituximab era, its significance and clinical relevance to contemporary clinical practice is questioned. 

To address the role of auto-HCT versus salvage chemotherapy alone in the rituximab-era, Sebban et al. conducted a *post hoc* analysis of patients enrolled on two GELF (Groupe d'Etude des Lymphomes Folliculaires) protocols that subsequently relapsed and received various salvage therapies including auto-HCT. In patients who received rituximab-containing salvage therapies, no statistically significant EFS or OS benefits were reported after HDT and auto-HCT when compared to patients who did not undergo autografting [28]. In a different study, the combined retrospective data from DFCI and St. Bartholomew's Hospital suggested prolonged remissions in a subset of FL patients after HDT; however, this benefit appeared restricted mostly to patients in second CR [29]. Conceptually, HDT and autologous transplantation at such an early point in relapsed FL could be uniformly offered, if it was curative and devoid of long-term serious complications. Auto-HCT, unfortunately, cannot be considered a curative modality for majority of the patients with FL. Large registry data from EBMT [30] and Center for International Blood and Marrow Transplant Research (CIBMTR) show no plateau in risk of disease relapse after autografting [31]. More importantly the risks of second cancers and sMDS/AML after auto-HCT are not insignificant, ranging from 5 to 15% in several large studies [29, 30]. While acknowledging the limitations of HDT in relapsed FL, it is also prudent to highlight the fact about a third of carefully selected chemosensitive FL patients that can experience durable responses following auto-HCT (31% PFS at 10 years in the EBMT registry data) [30].

In order to solve the problem of autograft contamination by lymphoma cells, several studies have examined the role of *ex vivo* purging (by monoclonal antibodies, CD34+ cell selection, etc.) [32, 33] and *in vivo* purging (e.g., rituximab with mobilization) [34, 35] of autologous stem cell products with encouraging results. However, the lack of randomized data to prove the superiority or curative potential of purged auto-HCT [27], and a possible increase in infectious complications with *ex vivo* purging [36, 37], has prevented the uniform acceptance to this modality by transplant centers. In the rituximab era, the decision to offer an autologous transplant should take into account several factors including patient's age, associated comorbidities, risk of secondary cancers, and presence of chemosensitive disease. Heavily pretreated patients with refractory disease are unlikely to benefit from HDT and should preferably be considered for participation in clinical trials. Outside the setting of a clinical trial, the decision to offer an auto-HCT for FL should be made on a case-by-case basis. Auto-HCT is best reserved for chemosensitive, relapsed FL patients after 2-3 lines of prior chemoimmunotherapies (ideally at least one doxorubicin-based line, and a bendamustine-based regimen), who are not candidates for curative therapies, namely, allo-HCT, because of donor unavailability, associated comorbidities, or patient preference. Whether postauto-HCT rituximab maintenance will improve patient outcomes is an area of active investigation and at the moment, it cannot be considered a standard option [38, 39].

### 3.2. Myeloablative Allogeneic Transplantation for Relapsed FL

Adoptive immunotherapy in the form of allo-HCT is potentially curative for patients with FL. The GVL effects mediated by the donor T-lymphocytes are beneficial in patients with lymphoid malignancies [40, 41]. One of the most compelling evidence for a clinically relevant GVL effect in relapsed FL comes from the success of allo-HCT after an autograft failure [42–44]. Unlike auto-HCT where relapse-risk posttransplant does not decrease overtime, registry data from CIBMTR and EBMT [31, 45] clearly show that a plateau in relapse risk is achievable in 2-3 years after allografting, indicating that a substantial proportion of these patients can be cured with MA allo-HCT. However, in both CIBMTR and EBMT studies, despite impressively low relapse rates (20–25% at 5 years) after MA allo-HCT, compared to rates following auto-HCT (50–55% at 5 years), no difference in OS was seen, primarily due to unacceptably high rates of TRM following MA allografts (approximately 35–40% compared to 8–15% after auto-HCT). Moreover, since the median age at diagnosis for FL is the sixth decade of life, a significant number of such patients are not appropriate candidates for MA conditioning. Whether there is a benefit of MA allo-HCT in younger patients with chemorefractory disease, over less ablative, so-called RIC regimens, is not known. It is unlikely that a prospective clinical trial will be performed to compare MA conditioning with RIC allogeneic transplantation in patients with FL, as the latter has been broadly adopted as the preferred regimen to use when considering allografting. In the absence of robust prospective data to prove otherwise, MA allo-HCT should not be considered as the regimen of choice in patients with FL, especially for those with advanced age and/or with associated medical comorbidities and poor performance status. 

### 3.3. RIC Transplantation for Relapsed FL

RIC regimens were developed to improve applicability of allo-HCT to older, heavily pretreated patients, particularly those with associated medical comorbidities. These regimens aim at reducing procedure-related toxicities and rely more heavily on GVL immunologic effects. While no prospective trials have compared MA conditioning against RIC transplantation in FL, registry data from EBMT and CIBMTR, with their inherent limitations [31, 45], have established the feasibility of this approach by demonstrating acceptable rates TRM [46], albeit at the possible expense of higher relapse rates [47] and comparable OS and PFS with RIC allo-HCT compared to MA allografts.

Several phase II studies have prospectively assessed the feasibility of RIC HCT in patients with relapsed FL (Table 2) [42, 48–51]. Khouri et al. have recently reported updated M.D. Anderson Cancer Center (MDACC) experience with 47 chemosensitive FL patients conditioned with fludarabine, cyclophosphamide, and high-dose rituximab. The 11-year PFS and OS were 72% and 78%, respectively. The incidence of grade 2–4 acute GVHD was 11% [48, 52]. This updated report from MDACC also includes 26 patients (38% with chemorefractory disease) who received novel conditioning with ^90^Y-ibritumomab tiuxetan. The 3-year PFS rates for patients with chemorefractory and chemosensitive disease were 80% and 87%, respectively [52]. The Cancer and Leukemia Group B (CALGB) also reported encouraging outcomes of FL patients with RIC in a smaller, but multicenter prospective study [51]. The Blood and Marrow Transplant Clinical Trials Network (BMT CTN) Protocol 0701 is currently conducting a multicenter study using the RIC reported by Khouri et al. It is important to point out that the CALGB study and 2008 publication by Khouri et al. comprised almost exclusively of patients undergoing matched sibling donor HCT. To mitigate the higher rates GVHD associated with unrelated donor (URD) HCT, Thomson et al. employed *in vivo* T-cell depletion with alemtuzumab. In this large multicenter study, 52% of the patients underwent URD transplantation. Ten percent of cases had refractory disease. The 4-year rates of PFS, OS, and TRM were 76%, 76%, and 15%, respectively, with clinically significant acute GVHD noted in 13% [49]. Nevertheless, relapse rates were slightly high (26%) and donor lymphocyte infusions were frequently needed, likely because of the use of T-cell depletion.

### 3.4. Autologous versus RIC Allogeneic Transplant for Relapsed FL

A commonly encountered question in the clinic is whether to offer auto- or RIC allo-HCT to patients with FL relapsing after multiple lines of prior therapies. An adequately powered prospective trial comparing these two options is lacking [1]. Unfortunately, the very important BMT CTN 0202 trial comparing auto-HCT to RIC allo-HCT in FL was closed early due to poor accrual (*N* for auto-HCT = 22 and *N* for allo-HCT = 8) [53]. For the 30 patients enrolled in the BMT CTN 0202 study, the 3-year OS was 73% with auto-HCT versus 100% following allo-HCT, and 3 year PFS was 63% in the auto-HCT group versus 86% in the allo-HCT cohort. No patient had grade II–IV acute GVHD. Three auto-HCT recipients died from nonrelapse causes. The Canadian group recently reported 3-year PFS and OS of 96%, with a novel approach of auto-HCT followed by a tandem RIC allo-HCT, with low rate of TRM [54]. Whether a tandem auto/allo-HCT approach is truly superior to the current clinical practice of effective cytoreduction with chemoimmunotherapy followed by allo-HCT is not known, and at the present time a tandem auto/allo-HCT should be considered investigational. While acknowledging the scarcity of good quality clinical trial data, it appears that TRM rate with RIC allo-HCT [48, 49, 51] is relatively low, with much lower risk of disease relapse and no risk of sMDS/AML, when compared against auto-HCT. Considering these facts, it is appropriate to offer RIC allo-HCT for appropriately selected and clinically fit FL patients with an available suitable adult donor, when curative intent is pursued. While the timing remains controversial, we consider this option mainly in patients who have progressed after 2-3 lines of prior therapies (including at least one with anthracyclines and/or fludarabine), provided that the disease remains chemosensitive and patients are not candidates for clinical trials. Auto-HCT can be considered for patients who are medically unfit for RIC allografting or those without an adult or alternative donor, with the understanding that cure may not be achievable. 

## 4. Transplantation for Transformed FL

Histological transformation of FL (HT-FL) to aggressive NHL is not uncommon with up to 30% of FL patients undergoing transformation, at an annual rate of 3% [55]. Studies evaluating the role of HCT in this setting are limited by a small sample size and unavailability of prospective data.  Table 3 details selected studies evaluating auto-HCT for HT-FL, that involved at least 20 patients [56–60]. The EBMT reported the largest study, involving 50 patients, all with chemosensitive, disease. The 5-year PFS and OS rates were 30% and 51% respectively [61]. The Norwegian group recently published the only prospective trial of auto-HCT in HT-FL. This study showed 5-year PFS and OS rates of 32% and 47%, respectively, in 30 patients [60]. Short followup and patient selection (with majority of patients with minimal disease at transplantation) is a limitation to consider when interpreting these results. An often overlooked clinical problem in this setting is the possibility of developing late relapses, mostly involving the indolent histologic component after auto-HCT, indicating that while HDT might potentially eradicate the large cell component, the (nontransformed) FL component appears less curable in this setting. 

To circumvent this problem, and to salvage patients with chemorefractory disease, limited data is available for allo-HCT in HT-FL. One study evaluated the role of RIC in 16 patients with HT-FL and reported a dismal 3-year TRM, PFS, and OS rates of 43%, 21%, and 18% respectively [62]. A South African report, which included HT-FL patients (*n* = 11) who received MA conditioning, showed OS of 64% [63]. Hamadani et al. reported a 100-day TRM of 12% and 5-year PFS and OS of 56% and 66%, respectively, in a cohort of 8 HT-FL patients that included bulky disease (*n* = 3) and/or chemorefractory disease (*n* = 3) [59]. At this time, HT-FL is best managed within the context of a clinical trial. At our institution, elderly transformed patients with minimal disease are typically offered auto-HCT, while the younger patients with minimal comorbidities and those with refractory transformed disease are offered participation on an ongoing allo-HCT clinical trial (http://www.clinicaltrials.gov/, NCT01203020).

## 5. Conclusions

Table 4 documents recommendations on the role of HCT in FL based on aforementioned reviewed data and expert opinion [64]. FL is a heterogeneous disease entity with variable presentation and clinical course. Currently, no predictive clinical or molecular markers to guide role of HCT therapy exist and this issue remains an area of active research. With improvements seen in management of newly diagnosed FL, including immunochemotherapy and rituximab maintenance [21], HCT is unlikely to play a role in the frontline setting. In the relapsed setting, prospective cooperative group effort is certainly needed to elucidate the optimal timing and overall role of HCT. Ongoing clinical trials are assessing the role of rituximab, for *in vivo* purging prior to auto HCT (NCT00856245) and radio-immunotherapy for disease control in the peri-transplant period. Whether the encouraging, but limited, data of tandem autologous and RIC allo-HCT [54] in FL will play a role in future awaits confirmation with a randomized control study. For allo-HCT to become a more widely accepted curative modality for majority of FL patients it will require development of safer and less toxic conditioning regimens, more effective ways of augmenting the beneficial GVL without increasing the incidence and severity of GVHD, and improving supportive care measures after transplantation. The BMT CTN protocol 0701 (NCT00912223) is a step in the right direction, but the need remains for more robust randomized, clinical trials. While slow accrual has led to premature closure of several key clinical trials [53], continued cooperative efforts are necessary.

## Figures and Tables

**Table 1:** Randomized prospective trials addressing the role of autologous transplantation in follicular lymphoma patients in first remission.

Study group (year)	Number of patients	TRM in HDT versus C	EFS/PFS in HDT versus C (years)	OS in HDT versus C (years)	Comments
GLSG (2004)	307	<2.5% in both arms	64% versus 33%; *P* < 0.0001 [5]	Not reported	Significantly more sMDS/AML with HDT (3.5% versus 0%; *P* = 0.02)

GELA (2006)	402	Not reported	38% versus 28%; *P* = 0.11 [7]	76% versus 71%; *P* = 0.53 [7]	Secondary malignancy similar in both groups—14 with chemotherapy and 11 with HDT

GOELAMS (2009)	166	Not reported	64% versus 39%; *P* = 0.004 [9]	76% versus 80%; *P* = 0.55 [9]	Significantly more secondary malignancies with HDT (*n* = 12 versus 1; *P* = 0.01)

GITMO (2008)	136	*n* = 3 versus *n* = 2 at 100 days	61% versus 28%; *P* < 0.01 [4]	81% versus 80%; *P* = 0.96 [4]	4-year MDS/AML was higher with HDT (6.6% versus 1.7%)

Abbreviations: GLSG: german low grade lymphoma study group; GELA: groupe d'etude des lymphomes de l'adulte; GOELAMS: groupe ouest-est des leucémies et autres maladies du sang; GITMO: gruppo italiano trapianto di midollo osseo; TRM: treatment-related mortality; HDT: high-dose therapy and autologous HCT arm; C: chemotherapy arm; EFS/PFS: event/progression-free survival; OS: overall survival; sMDS: secondary myelodysplastic syndrome; AML: acute myeloid leukemia.

**Table 2:** Results of prospective, phase II trials evaluating allogeneic hematopoietic cell transplantation after reduced intensity conditioning.

Author (Year)	Number of patients	Age (range)	Conditioning regimen	TRM	EFS/PFS	OS	Comments
Khouri et al. [48, 52] (2008 and 2012)	47	53 (33–68)	FCR +/− ATG	15% (5 years)	72% (11 years)	78% (11 years)	Grades 2–4 acute GVHD in 11%. All had chemosensitive disease. High-dose rituximab (1000 mg/m^2^) used.

Thomson et al. [49] (2010)	82	45 (26–65)	FMC	15% (4 years)	76% (4 years)	76% (4 years)	Grades 2–4 acute GVHD in 13%. Included 26% with prior auto-HCT and 9% with refractory disease.

Pi$\tilde{\text{n}}$ana et al. [50] (2010)	37	50 (34–62)	FM	35%*	57% (4 years)	54% (4 years)	Grades 2–4 acute GVHD in 47%. Included 46% with prior auto-HCT.

Shea et al. [51] (2011)	44 (16 had FL)	53 (39–68)	FC	9% (3 years)	75% (3 years)	81% (3 years)	All were sibling donors and none had prior auto-HCT.

Abbreviations: FL: follicular lymphoma; FCR: fludarabine, cyclophosphamide, rituximab; ATG: antithymocyte globulin; F: fludarabine, M: melphalan, C: campath; FC: fludarabine, cyclophosphamide; TRM: treatment-related mortality; EFS/PFS: event/progression-free survival; OS: overall survival; GVHD: graft versus host disease; auto-HCT: autologous hematopoietic cell transplantation. *TRM estimated from numbers in the publication.

**Table 3:** Autologous hematopoietic cell transplantation for follicular lymphoma that has undergone histological transformation to large cell lymphoma.

Author (year)	Number of patients	Age (range)	Conditioning regimen	TRM	PFS	OS	Comments
Friedberg et al. [56] (1999)	21	44 (29–58)	TBI/CY	NA	46% (5 years)	58% (5 years)	All had minimal disease state. Purged autograft used.

Chen et al. [58] (2001)	25^a^	48 (36–64)	Mel/TBI/VP	28%	36% (5 years)	37% (5 years)	All had chemosensitive disease.

Williams et al. [57, 61] (2001)	50	40 (26–52)	Various regimens	8% (100 days)	30% (5 years)	51% (5 years)	100% had chemo-sensitive disease. High LDH led to poor outcomes.

Hamadani et al. [59] (2008)	24	56 (47–68)	BU/CY BCNU based	8% (100 days)	40% (3 years)	52% (3 years)	17% had bulky disease and no purged autografts used.

Eide et al. [60] (2011)	30^b^	55 (31–65)	BEAM	NA	32% (5 years)	47% (5 years)	The only prospective trial. All 30 had chemosensitive disease.

Abbreviations: TBI: total body irradiation; CY: cyclophosphamide; Mel: melphalan; VP: etoposide; BU: busulfan; BCNU: carmustine; TRM: treatment-related mortality; PFS: progression-free survival; OS: overall survival; LDH: lactate dehydrogenase; BEAM: BCNU, etoposide, cytarabine, melphalan.

^a^Of the 35 patients in the sample, only 25 had true histological transformation to diffuse large B-cell lymphoma. ^b^Of the 47 patients enrolled, only 30 underwent autologous hematopoietic cell transplantation.

**Table 4:** Recommendations based on current evidence and expert opinion, on the role of hematopoietic cell transplantation in follicular lymphoma.

Status of FL	Type of HCT	Recommendations
First remission as consolidative therapy	HDT-autologous HCT	Not recommended.
	Allogeneic HCT	Not recommended.

	HDT-autologous HCT	Consider for patients with chemosensitive disease, and ≤2-3 lines of prior therapies.
Relapsed/refractory FL	Myeloablative allogeneic HCT	Best reserved for medically fit younger patients with refractory disease.
	RIC allogeneic HCT	Recommended for appropriately selected relapsed/refractory patients.

FL after histological transformation	HDT-autologous HCT	Appropriate for patients with chemosensitive disease. Ideally on a clinical trial.
	Allogeneic HCT	Consider for fit patients with refractory relapse, bone marrow involvement, and history of prior autologous HCT. Ideally on a clinical trial.

Abbreviations: FL: follicular lymphoma; HCT: hematopoietic cell transplantation; HDT: high-dose therapy; RIC: reduced intensity conditioning; RCT: randomized controlled trials; OS: overall survival; PFS: progression-free survival; TBI: total body irradiation; TRM: treatment-related mortality; URD: unrelated donor.
